# HIV testing and its associated factors among street-based female sex workers in Iran: results of a national rapid assessment and response survey

**DOI:** 10.1186/s13011-021-00382-x

**Published:** 2021-05-17

**Authors:** Sina Ahmadi, Mehrdad Khezri, Payam Roshanfekr, Salah Eddin Karimi, Meroe Vameghi, Delaram Ali, Elahe Ahounbar, Mehdi Noroozi, Mostafa Shokoohi

**Affiliations:** 1 Social Welfare Management Research Center, University of Social Welfare and Rehabilitation, Sciences, Tehran, Iran; 2 HIV/STI Surveillance Research Center, and WHO Collaborating Center for HIV Surveillance, Institute for Futures Studies in Health, Kerman University of Medical Sciences, Kerman, Iran; 3 Social Determinants of Health Research Center, Tabriz University of Medical Sciences, Tabriz, Iran; 4 Substance Abuse and Dependence Research Center, University of Social Welfare and Rehabilitation Sciences, Tehran, Iran; 5 Social Determinants of Health Research Center, University of Social Welfare and Rehabilitation Sciences, Tehran, Iran; 6 Dalla Lana School of Public Health, University of Toronto, Toronto, ON Canada

**Keywords:** Female sex workers, HIV testing, Drug use, Iran

## Abstract

**Background:**

Female sex workers (FSWs) are at a disproportionate risk of sexually transmitted infections and they may face significant barriers to HIV testing. This study aimed to examine HIV testing prevalence and its associated factors among street-based FSWs in Iran.

**Method:**

A total of 898 FSWs were recruited from 414 venues across 19 major cities in Iran between October 2016 and March 2017. Eligible FSWs were women aged 18 years of age who had at least one commercial sexual intercourse in the previous year. HIV testing was defined as having tested for HIV in the lifetime. Bivariable and multivariable logistic regression were used to examine the correlates of HIV testing. We report adjusted odds ratios (aOR) and their 95% confidence intervals (CI).

**Result:**

Overall, 57.8% (95%CI: 20.0, 88.0) of participants reported having tested for HIV, and HIV prevalence among FSWs who tested for HIV was 10.3% (95%CI: 7.5, 13.0). The multivariable model showed that unstable housing (aOR: 8.86, 95%CI: 2.68, 29.32) and drug use (aOR: 3.47, 95%CI: 1.33, 9.06) were associated with increased likelihood of HIV testing. However, FSWs with a higher level of income were less likely to be tested for HIV (aOR: 0.09, 95%CI: 0.02, 0.43).

**Conclusion:**

Almost one in ten street-based FSWs had never tested for HIV. These findings suggest the need for evidence-based strategies such as outreach support and HIV self-testing to improve HIV testing in this marginalized population.

## Background

While HIV in the general population is decreasing [1], various studies have shown that HIV infection is expanding among the high-risk and key populations [2, 3]. Female sex workers (FSWs) are known to be among such high-risk groups. A global study in low-income and middle-income countries estimated that the odds of HIV is 13.5 times higher among FSWs compared to women in the general population [2, 4]. The reasons offered for the increased risk of HIV infection among FSW are high-risk practices related to sex work, including condomless sex, multiple sexual partnerships, and drug use [2, 5].

In Iran, it is estimated that 228,700 women were engaged in sex work and FSWs have been recognized as the second most at-risk subpopulation for HIV infection [6]. In Iran, sex work is criminalized and exceedingly stigmatized [7]. Despite this, studies in Iran indicate that FSWs are at increased vulnerabilities to some health and behavioural outcomes. For example, a recent systematic review and meta-analysis in Iran estimated the pooled prevalence of HIV among FSWs as 2.23% [8]. Unsafe behavioural practices are also common among FSWs in Iran. A recent systematic review estimated the pooled prevalence of recent non-injection and injection drug use at 56.9 and 5.6%, respectively [9]. Observational studies showed that only 33.6 and 17.3% of FSWs reported consistent condom use with paying and nonpaying sex partners, respectively [10, 11].

Previous studies reported that less than one-third of FSWs in Iran get an HIV test [12], which could be due to concerns such as getting isolated or socially stigmatized [12–14]. Several studies have been demonstrated that there is a correlation between getting an HIV test and HIV risk perception, awareness of HIV test, age at sex work initiation, and received free of charge harm reduction services [14, 15]. To monitor the health status of this population and to better prevent transmission of HIV to other people, in particular, paying and non-paying partners, getting tested for HIV infection is highly important and recommended. Nevertheless, stigma against FSWs, low-risk perception, fear and worries, poor accessibility to HIV testing sites and services, and health providers’ reluctance to offer the test are the most common barriers towards HIV testing practices among FSWs in Iran and elsewhere [4, 16].

Despite the availability and expansion of free-of-charge HIV testing and referral in Iran, still few women tend to getting tested for HIV [4, 14]. In the present study, we aimed to estimate the prevalence of HIV testing and its associated factors among street-based FSWs in Iran. This is important to better understand the barriers of HIV testing and therefore for developing relevant programs to improve HIV testing among this population.

## Methods

### Study design and sampling

In this cross-sectional study, we recruited 898 FSWs from 414 venues between October 2016 and March 2017 in 21 major cities (21 provinces). The survey’s primary aim was to perform a rapid assessment of the situation of HIV-related risk behaviours among street-based FSWs in Iran [17, 18]. Through the venue-based application of time location sampling (TLS) method (i.e., spaces or locations are venues attended by the target population; times refer to specific days and periods when the target population congregates in each space), these spaces and associated days were categorized into standard space-time segments (four-hour intervals in each space) and are referred to as venue-day-time (VDT) units. Venues were initially identified by researchers who interviewed key informants and local experts in the qualitative phase of the study. The venues were chosen based on their population size and locations after our team visited all reported places. We used the random sampling method to select venues and recruit FSWs. A convenience sample of 3–5 eligible participants was recruited at each venue. Inclusion criteria were the women age 18 years or more who had at least one commercial sexual intercourse within the last 12 months preceding the interview. Verbal consents were obtained from eligible participants.

### Instruments

The questionnaire was in Farsi and included information on demographic characteristics, history of sex work, related sexual practices, and drug use. The survey questionnaire was based on previous questionnaires developed for HIV surveillance among FSWs in Iran [19, 20]. Data were collected using this standardized questionnaire and completed through face-to-face interview by trained staff.

### HIV testing

The current analysis explores the correlates of a lifetime HIV testing among FSWs through a self-reported history of having ever tested for HIV. Lifetime tested for HIV was treated as a binary variable and the responses to the question “Have you ever tested for HIV?” were coded as yes or no.

### Covariates

Potential exploratory factors of HIV testing included age at the time of interview (18–24, 25–34, or > 35 years old), level of education (never attended school, primary school, junior high school, or high school), marital status (single, married, divorced, temporary marriage [called: Sigheh], or widow), living status (with family or spouse, living alone, live in welfare center, homeless or live in shelters), history of abortion (yes or no), history of selling sex in a brothel (yes or no), self-perceived risk of HIV (yes or no), and HIV knowledge (low, moderate, or high). Participant’s HIV knowledge was measured using 14 questions with yes, no, or don’t know response options (e.g., a healthy-looking individual can have HIV, condoms can prevent HIV transmission, mosquito bites can transmit HIV, sharing meals with a person living with HIV can transmit HIV, and restricting sexual relationships to only one faithful but the uninfected partner can prevent HIV transmission). Participants who provided at least ten correct answers to these questions were defined as having a higher level of HIV knowledge (“sufficient”) vs. moderate and lower knowledge (“insufficient”).

### Statistical analysis

We first examined associations between variables and lifetime HIV testing using the chi-square or Fisher’s exact tests, as appropriate. Bivariable and multivariable logistic regression models characterized correlates of HIV testing. Variables with a *p*-value < 0.2 in the bivariable analysis were entered into a full multivariable regression model. Adjusted odds ratio (aOR) along with 95% confidence interval (CIs) were reported. Stata version 11 (Stata Corp.) was used for the statistical analysis.

## Results

The results showed that 57.8% (95%CI: 20.0, 88.0) of participants reported having tested for HIV, and HIV prevalence among FSWs who tested for HIV was 10.3% (95%CI: 7.5, 13.0). Higher prevalence of HIV testing was reported among FSWs who were in the 30–45 age category (62.0% vs. 9.1% for those aged > 46), were divorced (50.1% vs. 2.4% for those were single), earned between 100,000 and 500,000 Toman per month (34.2% vs. 16.7% for those earned between 1,000,000 and 5,000,000), and lived with family or spouse (58.5% vs. 5.4% for those lived in social welfare center). The prevalence of HIV testing was also higher among FSWs who reported ever drug use (81.0% vs. 18.9%), and current drug use (75.4% vs. 24.5%), perceived themselves to be at risk for HIV (74.9% vs. 25.0%), had sufficient knowledge of HIV (60.8% vs. 14.6%) and had not a history of abortion (54.5% vs. 45.4%) (Table 1).

**Table 1:** HIV testing among different subgroups of female sex workers in Iran’s first rapid assessment and response survey in 2017

Characteristics	HIV testing		***P***-value
	Yes (*n* = 495)	No (*n* = 361)	
	N (%)	N (%)	
**Age**			
18–29	137 (39.71)	139 (28.84)	0.001
30–45	193 (55.94)	299 (62.03)	
> 46	15 (4.35)	44 (9.13)	
**Education**			
Illiterate	24 (6.86)	33 (6.65)	0.091
Reading writing levels	12 (3.43)	38 (7.66)	
Primary education	62 (17.71)	102 (20.56)	
Secondary education	101 (28.86)	128 (25.81)	
High school	65 (18.57)	87 (17.54)	
Diploma	56 (16.00)	81 (16.33)	
College	30 (8.57)	27 (5.44)	
**Marital status**			
Single	4 (1.60)	10 (2.42)	0.006
Married	51 (20.40)	95 (23.00)	
Divorced	140 (56.00)	207 (50.12)	
Temporary marriage	18 (7.20)	63 (15.25)	
Widow	37 (14.80)	38 (9.20)	
**Living status**			
With family or spouse	208 (64.40)	271 (58.53)	0.036
Social welfare center	8 (2.48)	25 (5.40)	
Alone	48 (14.86)	57 (12.31)	
Homeless or shelter	59 (18.27)	110 (23.76)	
**Income per month**			
Under 100,000 T	20 (6.51)	80 (17.90)	< 0.001
100,000–500,000 T	92 (29.97)	153 (34.23)	
500,000-1,000,000	106 (34.53)	139 (31.10)	
1,000,000-5,000,000	89 (28.99)	75 (16.78)	
**Ever drug use**			
Yes	162 (48.50)	368 (81.06)	< 0.001
No	172 (51.50)	86 (18.94)	
**Current drug use**			
Yes	142 (85.54)	279 (75.41)	0.008
No	24 (14.46)	91 (24.59)	
**Ever injection**			
Yes	20 (11.76)	56 (15.34)	0.270
No	150 (88.24)	309 (84.66)	
**Last-month injection**			
Yes	14 (70.00)	19 (37.25)	0.013
No	6 (30.00)	32 (62.75)	
**HIV knowledge**			
Low	70 (19.39)	73 (14.66)	< 0.001
Moderate	168 (46.54)	122 (24.50)	
High	123 (34.07)	303 (60.84)	
**Self-perceived risk of HIV**			
No risk	72 (33.96)	89 (25.07)	0.023
At risk	140 (66.04)	266 (74.93)	
**Ever worked in brothel**			
No	130 (37.04)	177 (37.11)	0.984
Yes	221 (62.96)	300 (62.89)	
**Lifetime abortion**			
No	228 (64.96)	262 (54.58)	0.003
Yes	123 (35.04)	218 (45.42)	

The final multivariable model showed that living in a shelter or being homeless (aOR: 8.86, 95%CI: 2.68, 29.32) and drug use (aOR: 3.47, 95%CI: 1.33, 9.06) increased the likelihood of HIV testing. On the contrary, HIV testing was also less likely to be reported by FSW who had a higher level of income vs. those FSW with a lower level of income (aOR: 0.09, 95%CI: 0.02, 0.43) (Table 2).

**Table 2:** Bivariable and multivariable correlates of HIV testing among street-based female sex workers in Iran, 2017

Variables	HIV testing			
	Yes	No	Bivariable	Multivariable
	n (%)	n (%)	Crude OR (95%CI)	AOR (95%CI)
**Age**				
18–29	139 (28.84)	137 (39.71)	1	1
30–45	299 (62.03)	193 (55.94)	1.17 (0.82, 1.65)	1.70 (0.70, 4.13)
> 46	44 (9.13)	15 (4.35)	1.99 (0.86, 4.58)	1.34 (0.15, 12.03)
**Education**				
Illiterate	33 (6.65)	24 (6.86)	1	1
Reading writing levels	38 (7.66)	12 (3.43)	1.53 (1.03, 8.16)	2.02 (0.19, 21.00)
Primary education	102 (20.56)	62 (17.71)	1.15 (0.3, 2.50)	1.06 (0.18, 6.14)
Secondary education	128 (25.81)	101 (28.86)	0.71 (0.34, 1.49)	1.63 (0.36, 7.44)
High school	87 (17.54)	65 (18.57)	0.92 (0.43, 1.97)	1.85 (0.30, 11.37)
Diploma	81 (16.33)	56 (16.00)	0.96 (0.4, 2.11)	0.89 (0.17, 4.49)
College	27 (5.44)	30 (8.57)	0.46 (0.18, 1.19)	0.73 (0.07, 7.65)
**Marital status**				
Single	10 (2.42)	4 (1.60)	1	1
Married	95 (23.00)	51 (20.40)	0.56 (0.14, 2.19)	2.61 (0.31, 21.99)
Divorced	207 (50.12)	140 (56.00)	0.41 (0.10, 1.54)	1.22 (0.17, 8.56)
Patronage marriage	63 (15.25)	18 (7.20)	1.32 (0.31, 5.58)	3.45 (0.32, 36.41)
Widow	38 (9.20)	37 (14.80)	0.29 (0.07, 1.20)	6.55 (0.44, 96.56)
**Living status**				
With family or spouse	271 (58.53)	208 (64.40)	1	1
Social welfare center	25 (5.40)	8 (2.48)	2.26 (0.83, 6.10)	0.26 (0.05, 1.28)
Alone	57 (12.31)	48 (14.86)	1.14 (0.70, 1.85)	0.85 (0.27, 2.70)
Homeless or shelter	110 (23.76)	59 (18.27)	1.02 (0.65, 1.59)	8.86 (2.68, 29.32)
**Income (per month)**				
Under 100,000	80 (17.90)	20 (6.51)	1	1
100,000–500,000	153 (34.23)	92 (29.97)	0.30 (0.14, 0.62)	0.49 (0.12, 1.91)
500,000-1,000,000	139 (31.10)	106 (34.53)	0.28 (0.14, 0.59)	0.30 (0.06, 1.31)
1,000,000-5,000,000	75 (16.78)	89 (28.99)	0.20 (0.09, 0.43)	0.09 (0.02, 0.43)
**Current drug use**				
No	86 (18.94)	172 (51.50)	1	1
Yes	368 (81.06)	162 (48.50)	2.43 (1.37, 4.28)	3.47 (1.33, 9.06)
**Ever injection**				
Yes	56 (15.34)	20 (11.76)	–	–
No	309 (84.66)	150 (88.24)	–	–
**Self-perceived risk of HIV**				
No risk	89 (25.07)	72 (33.96)	1	1
At risk	266 (74.93)	140 (66.04)	1.80 (1.13, 2.86)	2.10 (0.76, 5.65)
**Lifetime abortion**				
No	262 (54.58)	228 (64.96)	1	1
Yes	218 (45.42)	123 (35.04)	1.41 (1.00, 1.98)	1.84 (0.81, 4.18)
**Ever worked in brothels**				
No	177 (37.11)	130 (37.04)	–	–
Yes	300 (62.89)	221 (62.96)	–	–

## Discussion

In this sample of street-based FSWs, we observed that approximately half of FSWs had never tested for HIV. Moreover, HIV testing was significantly associated with living in a shelter or being homeless, drug use, and lower-income. These findings suggest that despite scaling up free-of-charge HIV testing services and implementing interventions to increase the HIV testing uptake among key populations in Iran, HIV testing uptake is still low among these underserved women.

Our estimate for lifetime HIV testing in our FSWs sample falls within the range of other studies in Iran and elsewhere. For example, our estimate was relatively higher than the first national survey of FSWs in 2010 that indicated less than one-third of FSWs had ever tested for HIV and only one-fourth had tested for HIV in the past year [4, 15]. Our estimates for HIV testing are also higher than estimates for FSWs in other contexts [21, 22], and maybe it is because of sufficient information, the majority of women in this context consider themselves at risk of HIV because Iranian health policymakers have recognized FSWs’ healthcare and social needs [4]. In addition, information provision regarding HIV and its sequenced problems have caused people to become more knowledgeable and try to give blood HIV tests [12, 23]. However, HIV testing prevalence among our sample was lower than another study using data from the second national surveillance survey of FSWs reported more than two-thirds of their FSWs participants reported a recent HIV test result in 2015 [24]. Overall, the low HIV testing is concerning and requires scaling up innovative strategies to improve HIV testing among FSWs.

The findings of the multivariable analysis of the present study suggested that odds of getting examined for HIV among homeless or shelter resident FSWs were about eight times more than that in women who live with their spouses or families. This might be due to that the AIDS taboo is more right for women who live with their families than women who live in shelters. Also, FSWs who are homeless or live in shelters may engage in risky behaviors and experience more risk perception; these factors could be a motivator for them to get HIV tests. Moreover, our results suggest that getting an HIV testing is about three times higher among FSWs who use drugs compared to women who do not use drugs. One explanation for higher odds of HIV testing among FSWs who used drugs could be due to HIV testing offered in drug treatment settings. Previous studies reported that the probability of high-risk sexual behaviors decreases among FSWs who inject drugs compared to women who do not use drugs [25]; this might be due to the higher prevalence of HIV infection among them. Based on the findings, the odds for getting HIV tests among people with high income was 91% less than people with low income. This result was similar to other studies [26]. This may be because high-income FSWs may be more likely to use prevention strategies like condom use, fewer partners and therefore have a less risk perception of HIV infection.

Taken together, the study results suggest that approximately half of FSWs do not take an HIV test, while HIV prevalence is more among this population than other groups. This may be explained by insufficient information and the consequent low awareness of HIV testing sites and services among this group [23]. Studies suggested that most individuals from the high-risk groups were not aware that they could access free of charge HIV testing in Iran [4]; therefore, information provided in this area could impact taking HIV test among the high-risk groups, including FSWs. Additionally, while HIV rapid tests have been mainly provided as a part of counseling services and harm reduction settings in Iran, a growing body of evidence suggests that community-based HIV testing leads to higher HIV testing rates than facility-based testing [27, 28]. For example, evidence in Zambia demonstrated that HIV self-testing can address gaps in HIV testing coverage among FSWs [29]. Bringing community-based HIV testing such as HIV self-testing into current harm reduction and HIV prevention programs for FSWs in Iran is recommended to be implemented and evaluated. In addition, providing educational and stigma reduction programs to increase HIV testing knowledge and reduce the fear and worries associated with HIV testing through social media could promote HIV testing [24, 30].

We recognized the major limitations of our study. First, the study outcome was self-reported and may subject to social desirability and recall biases. Second, our sampling methods were not probability-based and therefore may not be generalizable. Third, associations reported in our study should not be interpreted as causal without careful consideration as our study was cross-sectional.

## Conclusion

HIV testing is low among street-based FSWs in Iran. Our findings underscore the need for developing evidence-based strategies to improve HIV testing uptake and perceptions of HIV risk. To do so, identifying the main barrier of HIV testing is highly important. The HIV prevention programs should consider developing novel and evidence-based programs to improve accessibility of HIV testing to FSWs, improve outreach services to better link trained communities with hard-to-reach groups, and reduce stigma associated with both sex work and HIV testing.

## Data Availability

The datasets used and/or analyzed during the current study are available from the corresponding author on reasonable request.

## Abbreviations

FSWs: Female sex workers
TLS: Time Location Sampling
VDT: Venue-day-time
aOR: Adjusted odds ratio
CI: Confidence interval
